# PDCA cycle reports: a quality tool to improve physician hand hygiene compliance

**DOI:** 10.1186/2047-2994-4-S1-P280

**Published:** 2015-06-16

**Authors:** P Gonçalves, JY Kawagoe, MFDS Cardoso, CV Silva, ADR Toniolo, HMF Castagna, CM dos Santos, FG de Menezes, L Correa

**Affiliations:** 1 Infection Prevention and Control Service, Hospital Israelita Albert Einstein, São Paulo, Brazil

## Introduction

Physician hand hygiene (HH) compliance is often found to be lower than that of nurses in the literature. In our institution HH compliance among physicians was about 40% despite many ongoing activities supported by WHO Multimodal HH Strategy to improve it. In 2013, a HH improvement PDCA (Plan-Do-Check-Act) category was created and the best works awarded at annual institutional Quality Exhibits.

## Objectives

Describe a strategy aimed at HH physician compliance improvement using a quality tool in 2013 and 2014.

## Methods

Descriptive study at a private institution with inpatient (650-beds) and six outpatient facilities, where voluntary and multidisciplinary groups developed HH improvement programs engaging patients and healthcare workers (HCWs) using a quality tool cycle – PDCA in 2013 and 2014. There were many strategies and metrics applied, including HH compliance, HH products consumption, HH knowledge and perceptions by HCWs and patients. For HH compliance there was a pre and post intervention measure.

## Results

In 2013, there were 30 PDCA works from outpatient and inpatient units/facilities, but 13 aimed at physician HH compliance. There were 1159 and 1393 opportunities and mean HH compliance of 35.2% and 54.6% pre and post intervention, respectively. In 2014 there were fewer PDCA works (19) presented and 6 aimed at physician HH compliance: 729 and 534 opportunities and mean HH compliance of 38.8% and 59.4% pre and post intervention, respectively. Engaging patients, HCWs and specially physicians was the main strategy used. The mean institutional HH compliance among physicians was 47.3% and 45.9% in 2013 and 2014 respectively.

## Conclusion

This strategy aimed at physician HH compliance improvement increased 55.1% and 53% in 2013 and 2014 respectively. Comparing HH compliance post intervention, the mean was higher in 2014, reaching 59.4%. The involvement and commitment of the team members as well as the managers and the coordinators was fundamental for the strategy successful.

## Disclosure of interest

None declared.
